# Understanding Tribofilm Formation Mechanisms in Ionic Liquid Lubrication

**DOI:** 10.1038/s41598-017-09029-z

**Published:** 2017-08-16

**Authors:** Yan Zhou, Donovan N. Leonard, Wei Guo, Jun Qu

**Affiliations:** 1 Materials Science and Technology Division, Oak Ridge National Laboratory, Oak Ridge, TN 37831 USA; 2 Center for Nanophase Materials Sciences, Oak Ridge National Laboratory, Oak Ridge, TN 37831 USA

## Abstract

Ionic liquids (ILs) have recently been developed as a novel class of lubricant anti-wear (AW) additives, but the formation mechanism of their wear protective tribofilms is not yet well understood. Unlike the conventional metal-containing AW additives that self-react to grow a tribofilm, the metal-free ILs require a supplier of metal cations in the tribofilm growth. The two apparent sources of metal cations are the contact surface and the wear debris, and the latter contains important ‘historical’ interface information but often is overlooked. We correlated the morphological and compositional characteristics of tribofilms and wear debris from an IL-lubricated steel–steel contact. A complete multi-step formation mechanism is proposed for the tribofilm of metal-free AW additives, including direct tribochemical reactions between the metallic contact surface with oxygen to form an oxide interlayer, wear debris generation and breakdown, tribofilm growth via mechanical deposition, chemical deposition, and oxygen diffusion.

## Introduction

Tribological interfaces are crucial in modern machinery, including but not limited to manufacturing equipment, transportation vehicles, and wind turbines. Since the surface morphology, material composition, and contact condition all change rapidly, various approaches have been used to study the interface phenomena: on-line monitoring^1–4^, periodic oil analysis^5, 6^, and off-line worn surface analysis^7^. Oil-miscible ionic liquids (ILs) have recently been reported as novel lubricant anti-wear (AW) additives providing effective wear reduction that is widely attributed to the formation of a protective tribofilm on the contact area^8–14^. At the same phosphorus concentration, ILs offer potentially superior wear protection^8^ with less adverse impact on the exhaust emission catalysts^15^ compared with the conventional zinc dialkyldithiophosphates (ZDDPs).

ZDDPs are believed to decompose and then self-react to deposit a tribofilm primarily composed of zinc and iron phosphates and oxides^16–18^. Such a tribofilm growth model is not directly applicable to ILs, because ILs do not self-supply metal cations. Hence, there is urgency in understanding the mechanisms governing the IL tribofilm growth.

Wear debris is a collection of materials removed from the contact area during the wear process and thus contains important ‘historical’ interface information that, however, is often overlooked in the literature. While some IL tribofilms have been well characterized^9, 11, 12, 19–22^, there is no published literature on the wear debris resulted from the IL lubricated contacts. Wear debris produced in ZDDP-containing oils had been examined^23–27^ and its possible involvement in the tribofilm was proposed based on metallic ions exchange driven by the chemical hardness principle^23, 25, 28^, with Zn^2+^ in zinc phosphate (tribofilm amorphous matrix) exchanged with Fe^3+^ in iron oxide (wear debris).

We investigated the tribofilms and wear debris particles generated in a steel–steel sliding contact lubricated by a base oil containing a phosphonium–phosphate IL. This particular IL was selected because of its superior anti-scuffing and wear protection compared with conventional ZDDP or amine-phosphate^8, 11, 19^. This study reveals the morphology, nanostructure, and composition of the wear debris, and correlates them with the tribofilm composition and evolution. The IL tribofilm formation is proposed as a multi-step process: direct tribochemical reactions between the metallic contact surface with oxygen to form an oxide interlayer, wear debris generation and breakdown, tribofilm growth via mechanical deposition, chemical deposition, and oxygen diffusion. Equilibrium eventually is reached between the tribofilm growth and wear. The revealed tribofilm formation mechanism is expected to shed light on the fundamental understanding of the AW nature of IL and the design of next-generation lubricant additives.

## Results

The wear volumes of the steel balls in the lubrication with and without IL additive were 3.4 ± 0.4  × 10^−4^ mm^3^ and 8.7 ± 2.0 × 10^−4^ mm^3^, respectively. Wear on the steel flats was not measurable in either case. The addition of 1% IL effectively reduced the wear rate by >60%.

### Tribofilm formed in the ionic liquid-containing oil

**Figure 1:**
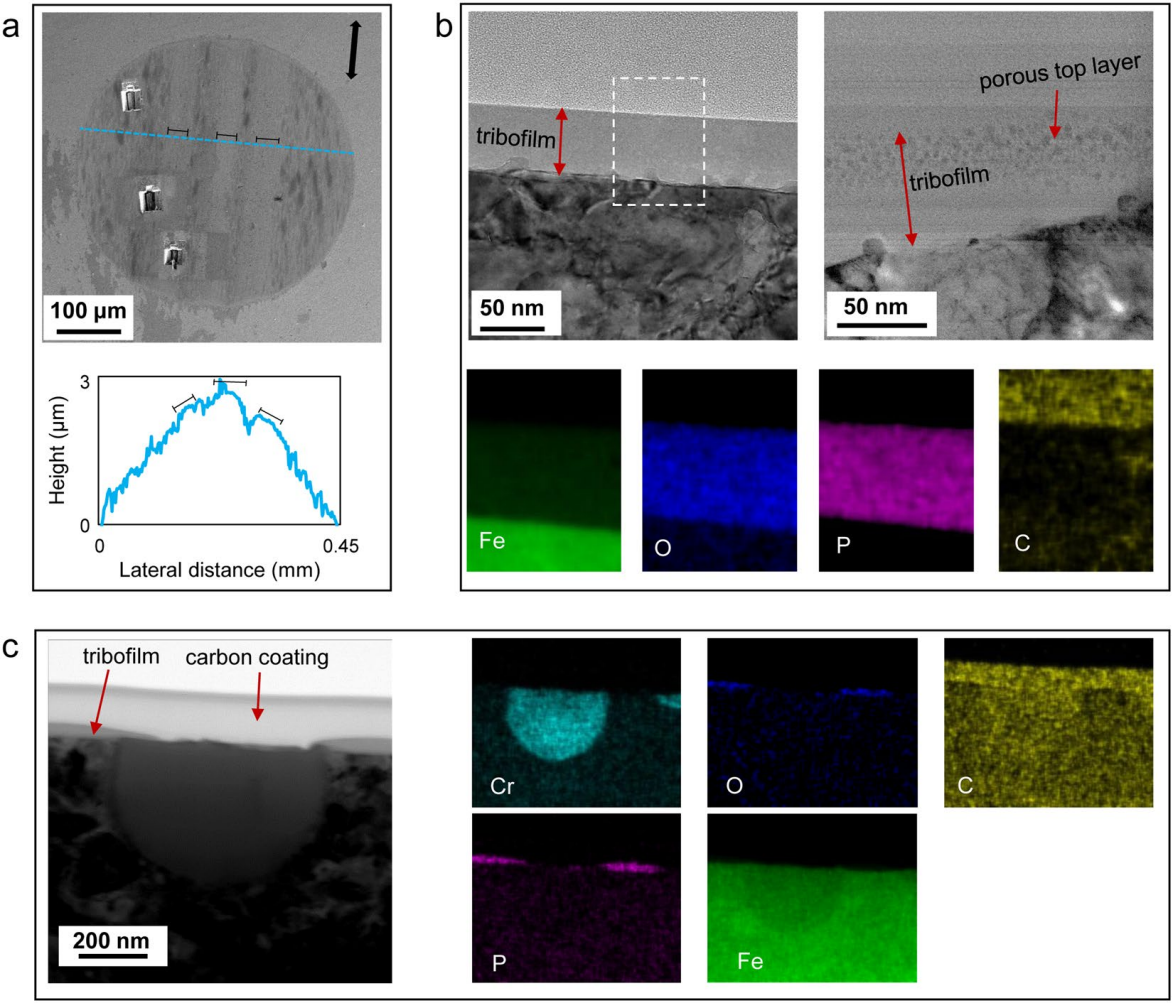
Morphological and compositional characterization of the worn surface on the steel ball lubricated by the base oil with 1% IL. (**a**) SEM image of the wear scar with a 2D profile; the arrow indicating the sliding direction and the three brighter stripes are marked (note: the height and lateral distance are not proportional). (**b**) Cross-sectional BF-STEM images of the tribofilm at two locations showing an amorphous structure of 50 nm and a two-layered structure (**a** porous top layer embedded with nanoparticles and an amorphous bottom layer); EDS elemental maps indicating the tribofilm is rich in Fe, O, and P. (**c**) A Cr grain with little tribofilm grew on top.

Tribofilms were generated on the contact areas during tribotesting. Figure 1a shows the wear scar on a steel ball that rubbed against a steel flat lubricated by the 0W-30 base oil containing 1% IL. The dark patches could indicate relative thick tribofilm pads. Along the sliding direction, there are three brighter stripes with possible thinner tribofilms. Based on the 2D profile, the brighter stripes are slightly taller than the rest of the worn surface, hence, are expected to experience a higher contact stress during the wear test and, as a result, to retain a thinner tribofilm. In Fig. 1b, the cross-sectional STEM images clearly present an amorphous tribofilm of 50 nm thick, and in some conditions, with a porous top layer composed of very fine nanoparticles. EDS elemental mapping suggests that the tribofilm is rich in Fe, O, and P, indicating a composite of iron oxides and iron phosphates based on the previous chemical analysis^21, 27–29^. A good bonding between the substrate and the bulk of a tribofilm is essential for its growth. Figure 1c shows a good example of the tribofilm which grew on top of the ferrous part of the steel substrate but not on a chromium grain. Analogous to oxidation behavior of stainless steels, the chromium rich region tends to form a passivation layer of chromium(III) oxide (Cr_2_O_3_) when exposed to oxygen, which prevents further reaction between ILs and Cr-rich substrate underneath.

**Figure 2:**
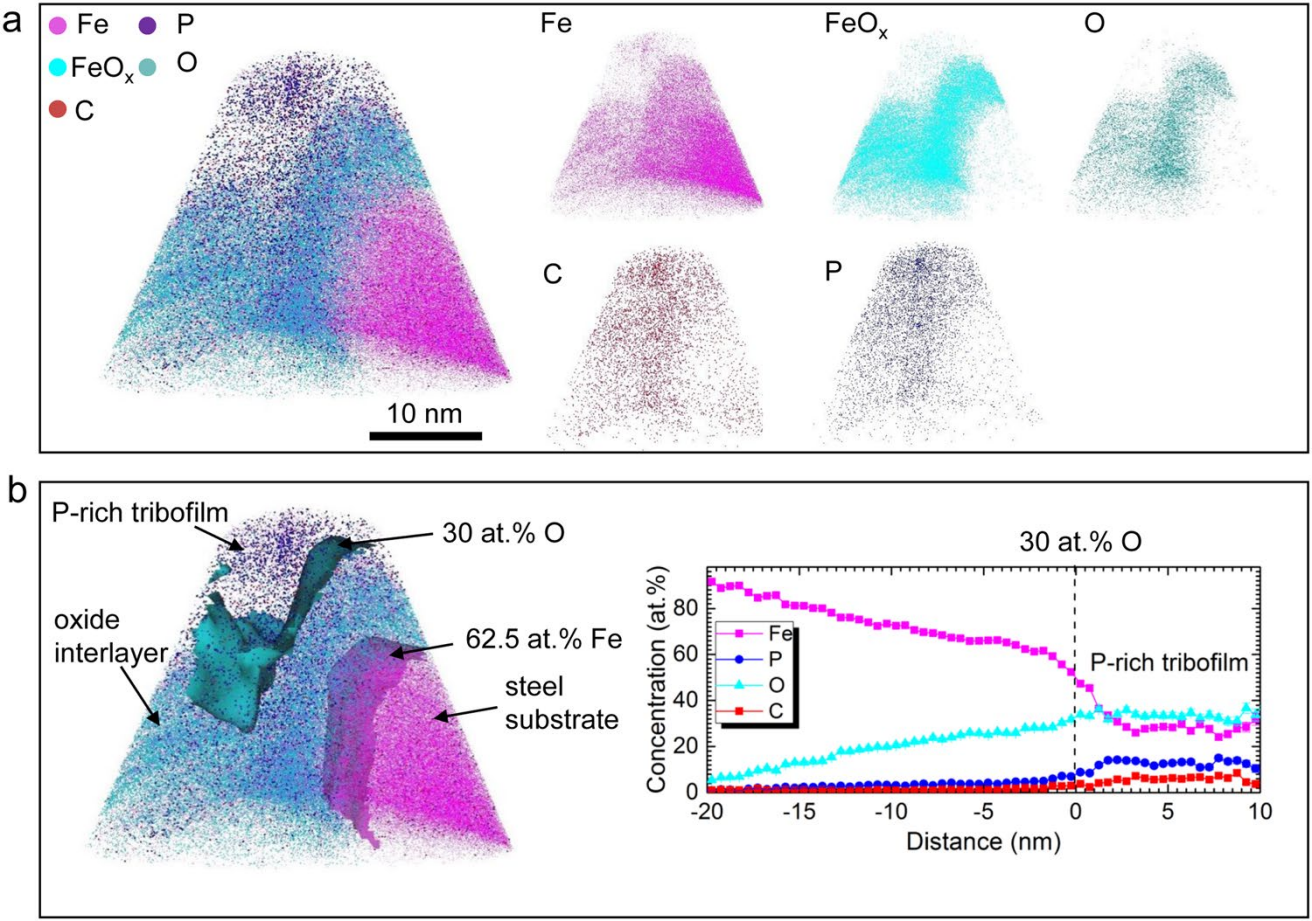
3D APT reconstruction of the IL tribofilm showing a gradient layered structure. (**a**) Spatial distribution of Fe, FeO_x_, O, C, and P ionic species. (**b**) Isoconcentration surfaces of 30 at.% O and 62.5 at.% Fe, and the proximity histogram analysis with respect to 30 at.% O isoconcentration surface.

The APT reconstruction of the IL tribofilm is shown in Fig. 2a, with each ion or ionic species represented in a single dot of an assigned color. The collected iron oxide ions are labeled as FeO_x_. In the atom probe map, an oxide interlayer appears between the P-rich tribofilm and the steel substrate. In Fig. 2b, two isoconcentration surfaces, i.e. 30 at.% O and 62.5 at.% Fe, were generated to categorize the layered structures. An oxide interlayer was found in between these two isosurfaces. The proximity histogram shows the composition evolution with respect to the distance away from the 30 at.% O isosurface. The P concentration in the tribofilm is as high as 15.0 ± 1.2 at.%.

### Wear debris produced in the ionic liquid-containing oil

**Figure 3:**
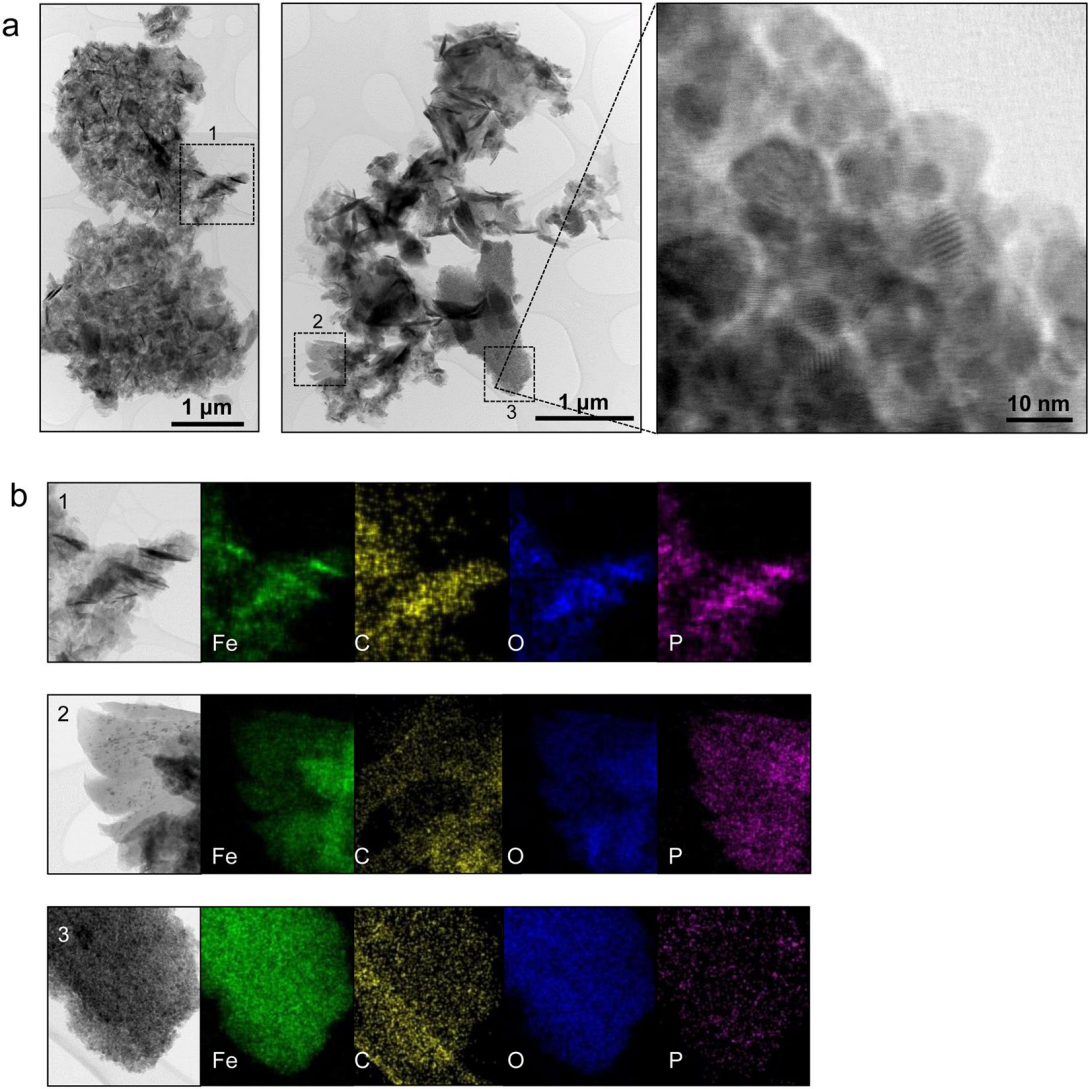
Wear debris collected from the steel–steel contact lubricated by the base oil with 1% IL. (**a**) BF-STEM images of the aggregations of wear debris particles, with a magnified view showing a cluster of nanoparticles. (**b**) EDS elemental maps of the three types of particles: (b-1) acicular, (b-2) flaky, and (b-3) fine spherical.

Figure 3a presents the BF-STEM images of aggregated wear debris generated from the IL-containing oil lubrication test. The aggregation could occur either during or post the tribotest. The wear debris particles appear to have different morphologies that are likely related to chemomechanical processes at the contact interface. There are three major types of wear debris particles: acicular (tens-hundreds nm long), flaky (tens-hundreds nm), and fine spherical (<10 nm). The acicular particles contain Fe, O, and P, indicating two possible routes of their origination and evolution: (i) removed tribofilm that contained iron oxides and phosphates and (ii) reaction products of metallic/metal-oxide debris with the IL. The flakey pieces were embedded with fine iron or iron oxide nanoparticles. This agrees with previous observations that the ZDDP wear debris flakes were embedded with iron or iron oxide nanoparticles^23, 30^. For the clusters of fine nanoparticles, EDS elemental mapping shows a high content of Fe and O but little P (Fig. 3b–3), suggesting oxidation to some extent. The square edges of the clusters hint that they might originally be spalled off the tribofilm surface where a top porous layer is present (see Fig. 1a) rather than produced in the lubricant at the contact interface.

### Worn surface and wear debris generated in the neat base oil

**Figure 4:**
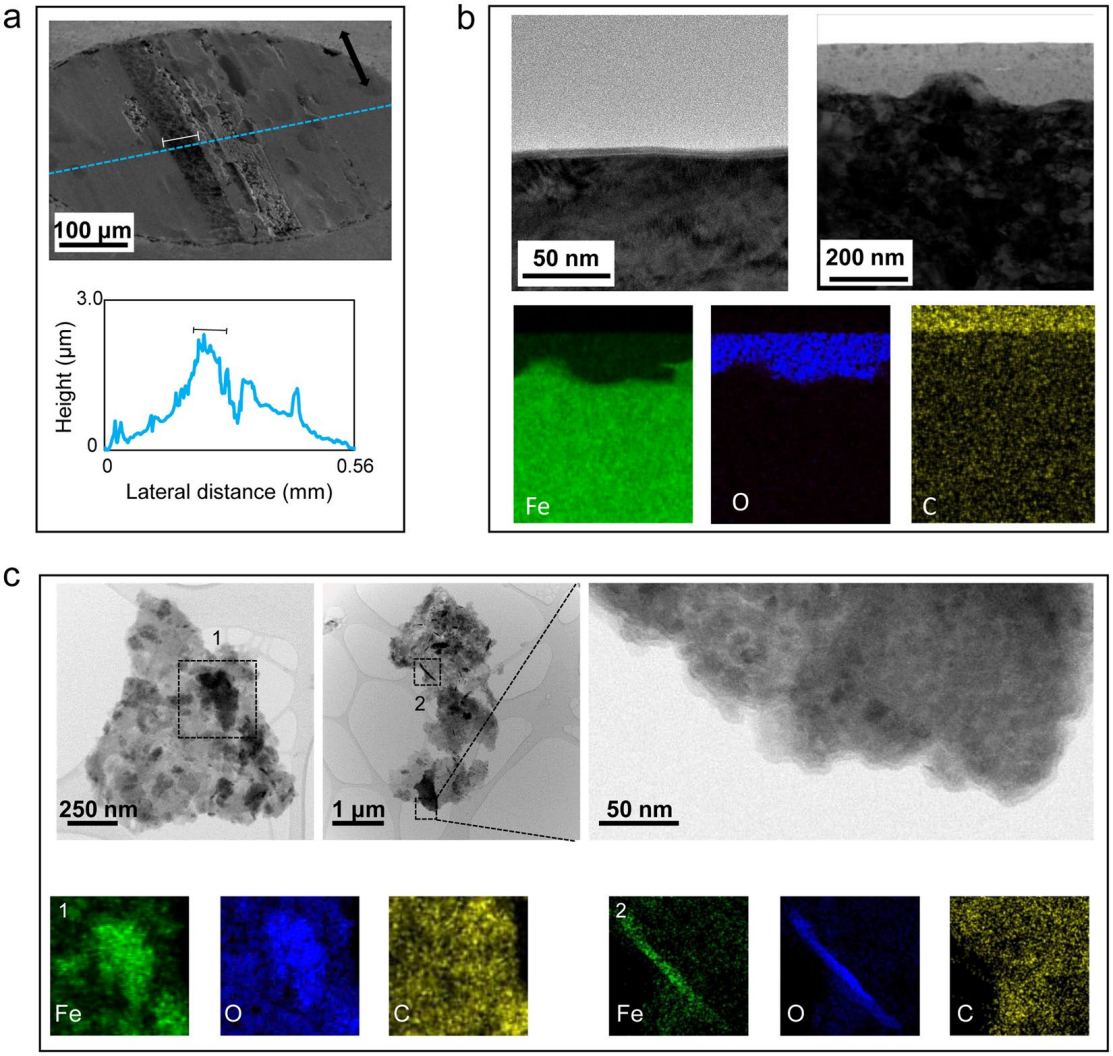
Worn surface on the steel ball lubricated by the neat base oil and the collected wear debris particles. (**a**) SEM image of the ball wear scar with a 2D profile (marked protrusion indicating adhesive material buildup). (**b**) Cross-sectional BF-STEM images of the worn surface showing a thin iron oxide film and an adhesive material buildup. (**c**) BF-STEM images of aggregated wear debris particles, with a magnified view of a cluster of fine nanoparticles. EDS elemental maps indicating mixed iron oxides and metallic iron.

For a direct comparison, the similar characterization was performed on the worn surface and wear debris produced in the 0W-30 base oil without any additives. This simple tribological system allows isolating any chemical complications induced by the IL. The SEM image (Fig. 4a) of the ball wear scar shows three distinctive regions: a central rough area, a dark stripe to the left, and the rest of the worn surface in a smoother appearance. The 2D profile displays grooving at the central rough strips and protrusion at the dark stripe. The central rough area was likely generated by a combination of two- and three-body abrasion, adhesion, and surface oxidation. The dark stripe endured a higher contact stress because of its protrusive nature and experienced an abundant supply of wear debris particles, especially from the adjacent exposed rough area, to cause a buildup of adhesive materials. In Fig. 4b, the cross-sectional STEM images exhibit a thin oxide film of a few nm and a layer of adhesive material buildup up to 200 nm thick with embedded nanoparticles, which were possibly wear debris being mechanically pressed in. The thin oxide film could be naturally occurring oxide that generated during storage. The EDS elemental maps indicate the composition of the surface layer is metallic iron and iron oxides.

The wear debris collected from the test in the neat base oil is shown in Fig. 4c. STEM images of the aggregations of wear debris containing clusters of fine spherical nanoparticles and acicular particles, similar to those observed in the wear debris from the IL-containing oil, except no flaky debris was found here. The EDS elemental mapping identified simply two major elements, Fe and O, suggesting a mixture of metallic iron and iron oxides.

## Discussion

Within the aggregations of wear debris particles, flakes were seen when the IL was present in the oil, while acicular and fine spherical particles were observed in both cases with and without the IL. When the steel flat was substituted with a much harder coating and in lubrication by the base oil plus 1% IL, the acicular and fine spherical particles were observed but the flakes were not, as shown in Fig. S1, probably due to a much severe grinding process that has broken the flakes apart.

### Flakey debris

The origin of the flakes is likely the amorphous tribofilm, whose “digestion” of small grain particles is responsible for the removal of hard and thus abrasive iron oxide particles—one of the antiwear mechanisms of ZDDP tribofilm^17^. The formation of flakey debris is believed to begin with the crack initiation mechanism upon external stress: the microcracks and voids formed around a single embedded nanoparticle or between particles, followed by surface decohesion between the particle surface and the amorphous matrix, and eventually a crack initiation that led to delamination. The flakes with embedded fine nanoparticles have also been observed in another ashless AW additive, acidic dialkyldithiophosphates, in a severe boundary lubrication regime^31^.

### Acicular debris

**Figure 5:**
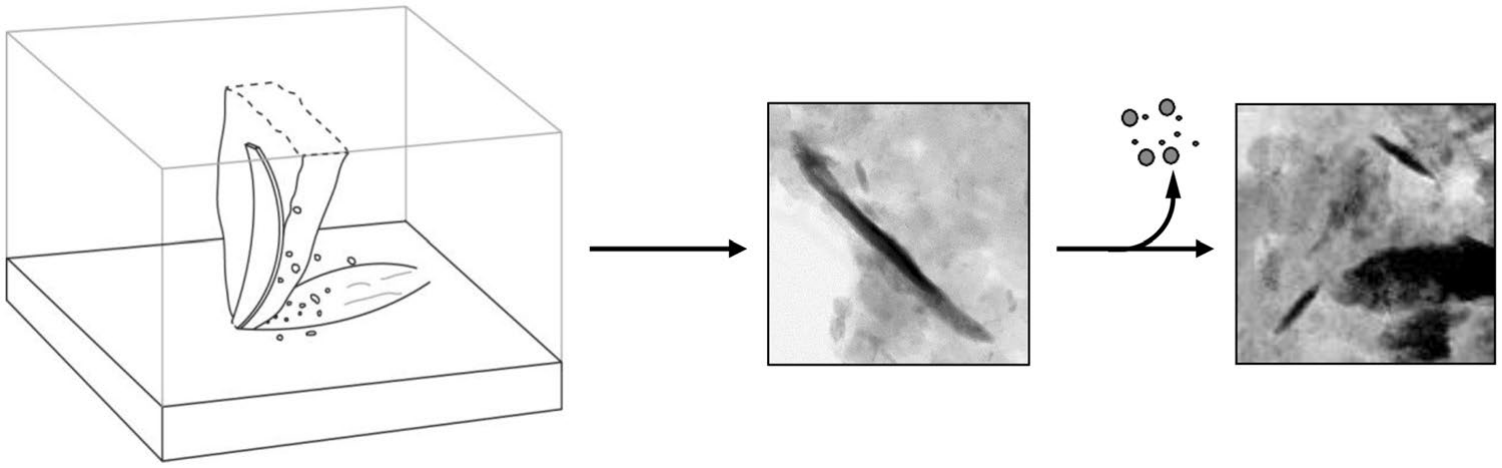
Schematic of an abraded acicular particle that is ground down.

The generation of the acicular-shape wear debris might require a sharp asperity contact, as hypothetically illustrated in Fig. 5. Upon contact and sliding, a sharp asperity penetrates and scratches the counterface, removing a piece of relatively large acicular material. The grinding process at the interface gradually breaks down the large acicular particles to smaller pieces with the edges and vertices rounded.

### Clusters of fine spherical nanoparticles

The fine nanoparticles could be either fractured surface asperities or ground larger pieces including the acicular debris. These fine nanoparticles aggregate to form clusters and may adhere to the contact area under pressure and later be spalled off. The fine nanoparticles could be the source of the porous top layer of the IL tribofilm as well as the thick buildup in base oil lubrication.

On a contact surface, the IL tribofilm is not homogenous, as seen in Fig. 1. It is believed that the tribofilm thickness and coverage are highly influenced by the surface local morphology and composition. The metal-free IL could share a similar mechanism with ashless extreme-pressure oil additives^29^. The tribofilm on the high plateaus, which experience relatively high normal and shear stresses, protects the substrate in a sacrificial manner. The material worn off the sacrificial layer is then incorporated into the wear debris pool and later deposited predominantly in the valleys where a thicker tribofilm forms.

**Figure 6:**
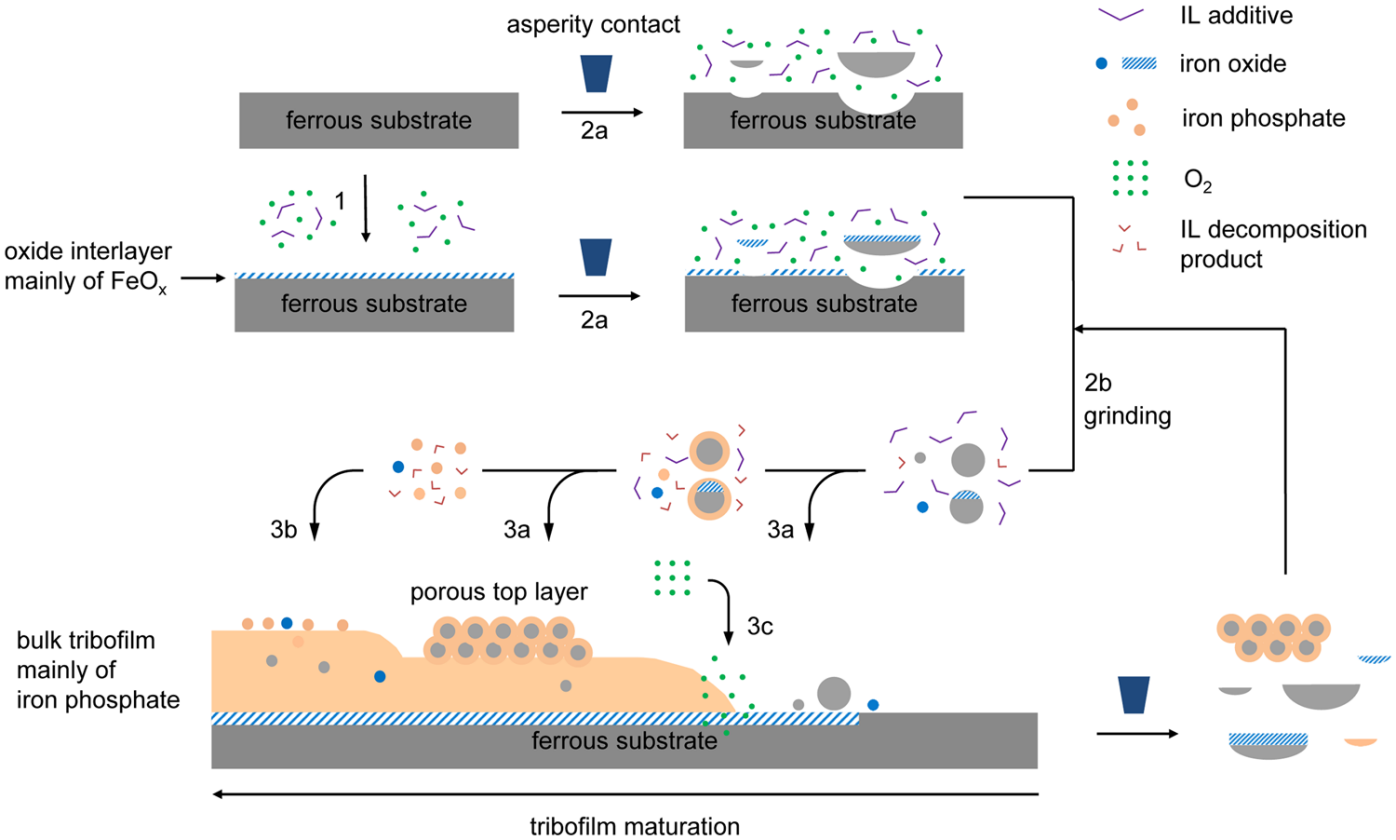
Proposed wear debris evolution and tribofilm growth for a ferrous substrate lubricated by an IL-containing lubricant. (1) Tribofilm initiation via direct surface reactions to generate an oxide interlayer mainly of iron oxide; (2) wear debris generation by (2a) asperity collisions, followed by (2b) breakdown through grinding process; (3) tribofilm growth via (3a) mechanical deposition, (3b) chemical deposition, and (3c) oxygen diffusion. Equilibrium eventually is reached between the tribofilm growth and wear.

Unlike the conventional ZDDP that self-reacts to grow a tribofilm, ILs require an external supply of metal cations, and the two apparent sources are the contact surface and the wear debris. Based on the characterization of tribofilms and wear debris, here we propose a complete multi-step formation mechanism for the IL tribofilm, as depicted in Fig. 6.

(1) Tribofilm initiation via direct surface reactions. The nascent ferrous surface reacts with oxygen to form a thin oxide interlayer. In Fig. 2, the APT composition mapping confirms the existence of such an iron oxides-based interlayer, which may provide a good bonding between the metal substrate and the tribofilm. On the other hand, the oxide interlayer could hinder the tribofilm growth by serving as a barrier between the metal and reducing surface defects for catalytic reaction sites^32^.

(2) Wear debris generation and breakdown. Upon asperity contact, material is removed from the nascent ferrous surface and the newly formed oxide interlayer. All the wear debris particles are included in the lubricant, with some trapped in the contact zone and others released to the outside. The grinding process at the contact zone breaks the large particles and removes their edges and vertices, hence gradually reducing the particle size.

(3a) Tribofilm growth via mechanical deposition. Wear debris particles may chemically react with reactive elements (oxygen, IL, and decomposition compounds of IL) upon the thermomechanical stresses at the contact interfaces. During contact and sliding, wear debris nanoparticles, reacted or not, are pressed onto the surface to form a porous top layer, as observed in Fig. 1 and in the literature^9, 12, 19, 22^. The top porous layer could spall off and re-enter the lubricant (Figs. 3a and 4c), or transform to the amorphous bulk tribofilm layer by mechanochemical reactions. Wear particles may also be incorporated inside the tribofilm through mechanical mixing, which was previously seen in severe boundary lubrication with excessive wear debris^9, 14^. Since the material removal in steel-steel contact is moderate, the inclusion of nanoparticles within the tribofilm was at minimum presence. In one case of steel rubbing cast iron, Fe_2_O_3_ nanoparticles can be clearly seen inside the tribofilm (Fig. S2) on the cast iron surface. This is a good example of wear debris embedded in the tribofilm, likely through mechanical mixing during the wear process.

(3b) Tribofilm growth via chemical deposition. The wear debris particles continue the mechanical breakdown and chemical reactions in the contact zone. The resulting iron oxide and iron phosphate compounds serve as precursors to nucleate on top of the tribofilm leading to further film growth.

(3c) Tribofilm growth via oxygen diffusion. Oxygen could potentially diffuse through the tribofilm and react with the metal substrate. The product from this reaction contributes to the growth of the oxide interlayer, but the increased film thickness is minor compared to the overall tribofilm.

Equilibrium eventually is reached between the tribofilm growth and wear. The growth of a tribofilm is inhomogeneous due to localized temperature, stress, material composition, and lubricant chemistry, etc. Phosphate cross-linking and phosphate–iron oxide attraction could be critical for the IL tribofilm integrity, but yet to be investigated.

## Conclusion

In conclusion, characterization was conducted on the wear debris and tribofilms generated in a steel–steel contact lubricated by a base oil with and without a phosphonium–phosphate ionic liquid. Results revealed the morphology, nanostructure, and composition of the wear debris, and correlated them with the corresponding tribofilms. We suggest a multi-step formation for the IL tribofilm, including direct surface reactions, wear debris generation and breakdown, mechanical deposition through pressing and mixing of wear debris, chemical deposition based on the nucleation of tribochemical reaction products between wear debris and reactive elements, and oxygen diffusion-facilitated oxide interlayer growth.

## Materials and Methods

Tribotests were conducted using a ball-on-flat configuration lubricated by a base oil without and with 1 wt.% of an IL, trihexyltetradecylphosphonium bis(2-ethylhexyl) phosphate ([P_66614_][DEHP]). This base oil is a mixture of Group III and IV base oils without containing any additive and is used for formulating an SAE 0W-30 engine oil (Chevron Corp., USA). The viscosity of this base oil is 26.6 cP at 23 °C, 13.8 cP at 40 °C, and 3.12 cP at 100 °C. The IL was synthesized following the procedure reported previously^9^. Tests were carried out on a reciprocating sliding tribometer (Plint TE77, Phoenix Tribology, UK) at a normal load of 100 N, an oscillation frequency of 10 Hz, a stroke of 10 mm, and a lubricant temperature of 100 °C. A 10-mm diameter grade 25 AISI 52100 bearing steel ball (*R*_a_: 0.025–0.05 μm) was used to slide against a flat of hardened M2 tool steel (*R*_a_: 0.06 μm). The initial Hertzian contact pressure is 2.19 GPa maximum and 1.46 GPa in average at the contact area. The minimum oil film thickness, calculated using the Hamrock and Dowson formula^33^, at the initial contact is 0.02 μm at the stroke center and near zero at the stroke ends. The λ-ratio (ratio of oil film thickness and composite roughness)^33^ is less than 0.3, indicating that the lubrication regime is boundary. The friction and wear results had been discussed in an early report, and this study focuses on the wear debris characterization. The wear volume was measured by using a 3D optical interferometer (Wyko NT9100). The wear volume on the steel flat was negligible and the depth of the ball wear scars was 1–2 orders of magnitude higher than the tribofilm thickness and thus the ball wear volume basically reflects the material loss. The tested wear debris particles were rinsed with isopropyl alcohol and then centrifuged to collect the precipitates. The washing and centrifugation process was repeated twice. The collected wear debris particles were deposited on a copper grid with a carbon layer.

The copper grid was examined using aberration-corrected scanning transmission electron microscopy (AC-STEM) and energy-dispersive X-ray spectroscopy (EDS). The AC-STEM instrument was a JEOL 2200 operated at 200 keV and equipped with a Bruker silicon drift detector (SDD) for X-ray analysis and elemental identification. Wear scars on the steel balls were imaged using scanning electron microscopy (SEM). Cross-sectional TEM samples of the tribofilms were prepared using focused ion beam (FIB). The process started with a carbon deposition of 50–60 nm in thickness on the surface to limit ion implantation into the tribofilm. A Hitachi NB5000 SEM/FIB system with a liquid gallium ion source was then used to extract a cross-section of the near surface zone. TEM bright field (BF) imaging and STEM/EDS of the lamella was performed at 300 keV using a Hitachi HF3300 instrument equipped with a Bruker Silicon Drift Detector (SDD) assembled EDS system. Atomic resolution STEM imaging was performed using an aberration-corrected Nion UltraSTEM operated at 100 keV. An FEI Nova 200 dual-beam FIB instrument was used to prepare the lift-outs of the specimen for atom probe tomography (APT). A wedge lift-out geometry was used to mount multiple samples on a Si micro-tip coupon to enable the fabrication of multiple APT needles from one wedge lift-out. The APT analysis was performed using a LEAP 4000X HR equipped with a pico-second 355 nm UV laser from CAMECA Instruments. The Integrated Visualization and Analysis Software (IVAS 3.6.12) from CAMECA Instruments was used to reconstruct the data.

## Electronic supplementary material

Supplementary Information

## References

[1] Blau PJ (2001). The significance and use of the friction coefficient. Tribol. Int..

[2] Al-Ghamd AM, Mba D (2006). A comparative experimental study on the use of acoustic emission and vibration analysis for bearing defect identification and estimation of defect size. Mech. Syst. Signal. Pr..

[3] Tonck A, Martin JM, Kapsa P, Georges JM (1979). Boundary lubrication with anti-wear-additives - study of interface film formation by electrical contact resistance. Tribol. Int..

[4] Glavatskih SB (2004). A method of temperature monitoring in fluid film bearings. Tribol. Int..

[5] Myshkin NK (2003). Wear monitoring based on the analysis of lubricant contamination by optical ferroanalyzer. Wear.

[6] Wang SS (2001). Road tests of oil condition sensor and sensing technique. Sensor. Actuat. B-Chem..

[7] Totten, G. E. & Liang, H. *Mechanical Tribology: Materials, Characterization, and Applications*. (CRC Press, 2004).

[8] Zhou Y, Qu J (2017). Ionic liquids as lubricant additives: a review. ACS Appl. Mater. Inter..

[9] Qu J (2012). Antiwear performance and mechanism of an oil-miscible ionic liquid as a lubricant additive. ACS Appl. Mater. Inter..

[10] Yu B (2012). Oil-miscible and non-corrosive phosphonium-based ionic liquids as candidate lubricant additives. Wear.

[11] Zhou Y (2014). Ionic liquids composed of phosphonium cations and organophosphate, carboxylate, and sulfonate anions as lubricant antiwear additives. Langmuir.

[12] Barnhill WC (2014). Phosphonium-organophosphate ionic liquids as lubricant additives: effects of cation structure on physicochemical and tribological characteristics. ACS Appl. Mater. Inter..

[13] Cai ZB (2014). Comparison of the tribological behavior of steel-steel and Si_3_N_4_-steel contacts in lubricants with ZDDP or ionic liquid. Wear.

[14] Barnhill WC (2016). Tertiary and quaternary ammonium-phosphate ionic liquids as lubricant additives. Tribol. Lett..

[15] Xie C (2016). Impact of lubricant additives on the physicochemical properties and activity of three‐way catalysts. Catalysts.

[16] Gosvami NN (2015). Mechanisms of antiwear tribofilm growth revealed *in situ* by single-asperity sliding contacts. Science.

[17] Spikes H (2004). The history and mechanisms of ZDDP. Tribol. Lett..

[18] Mosey NJ, Muser MH, Woo TK (2005). Molecular mechanisms for the functionality of lubricant additives. Science.

[19] Qu J (2014). Comparison of an oil-miscible ionic liquid and ZDDP as a lubricant anti-wear additive. Tribol. Int..

[20] Qu J (2015). Synergistic effects between phosphonium-alkylphosphate ionic liquids and zinc dialkyldithiophosphate (ZDDP) as lubricant additives. Adv. Mater..

[21] Zhou Y (2015). Does the use of diamond-like carbon coating and organophosphate lubricant additive together cause excessive tribochemical material removal?. Adv. Mater. Interfaces.

[22] Qu J (2015). Characterization of ZDDP and ionic liquid tribofilms on non-metallic coatings providing insights of tribofilm formation mechanisms. Wear.

[23] Martin JM (1999). Antiwear mechanisms of zinc dithiophosphate: a chemical hardness approach. Tribol. Lett..

[24] Varlot K (2000). Antiwear film formation of neutral and basic ZDDP: influence of the reaction temperature and of the concentration. Tribol. Lett..

[25] Martin JM (2012). The origin of anti-wear chemistry of ZDDP. Faraday Discuss..

[26] Mourhatch R, Aswath PB (2011). Tribological behavior and nature of tribofilms generated from fluorinated ZDDP in comparison to ZDDP under extreme pressure conditions-Part 1: Structure and chemistry of tribofilms. Tribol. Int..

[27] Mourhatch R, Aswath PB (2011). Tribological behavior and nature of tribofilms generated from fluorinated ZDDP in comparison to ZDDP under extreme pressure conditions-Part II: Morphology and nanoscale properties of tribofilms. Tribol. Int..

[28] Minfray C (2008). Experimental and molecular dynamics simulations of tribochemical reactions with ZDDP: zinc phosphate–iron oxide reaction. Tribol. T..

[29] Batchelor AW, Stachowiak GW (1986). Some kinetic aspects of extreme pressure lubrication. Wear.

[30] Martin JM, Mansot JL, Berbezier I, Dexpert H (1984). The nature and origin of wear particles from boundary lubrication with a zinc dialkyl dithiophosphate. Wear.

[31] Kim B, Mourhatch R, Aswath PB (2010). Properties of tribofilms formed with ashless dithiophosphate and zinc dialkyl dithiophosphate under extreme pressure conditions. Wear.

[32] Stachowiak, G. & Batchelor, A. W. *Engineering Tribology*. 409 (Elsevier Science, 2013).

[33] Hamrock, B. J. & Dowson, D. *Ball Bearing Lubrication: the Elastohydrodynamics of Elliptical Contacts*. (Wiley, 1981).

