# Supplementary material for: Understanding Tribofilm Formation Mechanisms in Ionic Liquid Lubrication
Source: Sci Rep. 2017 Aug 16;7:8426. doi: 10.1038/s41598-017-09029-z (PMC5559593; doi:10.1038/s41598-017-09029-z)
Supplement: Supplementary file 1 — Supplementary Information [file 41598_2017_9029_MOESM1_ESM.pdf]

Understanding Tribofilm Formation Mechanisms in Ionic Liquid Lubrication

Yan Zhou[1,], Donovan N. Leonard[1], Wei Guo[2], and Jun Qu[1,*]*

1. Materials Science and Technology Division, Oak Ridge National Laboratory, Oak Ridge, TN 37831, USA.

2. Center for Nanophase Materials Sciences, Oak Ridge National Laboratory, Oak Ridge, TN 37831, USA.

E-mail: zhouy@ornl.gov; qujn@ornl.gov

Supplementary Information

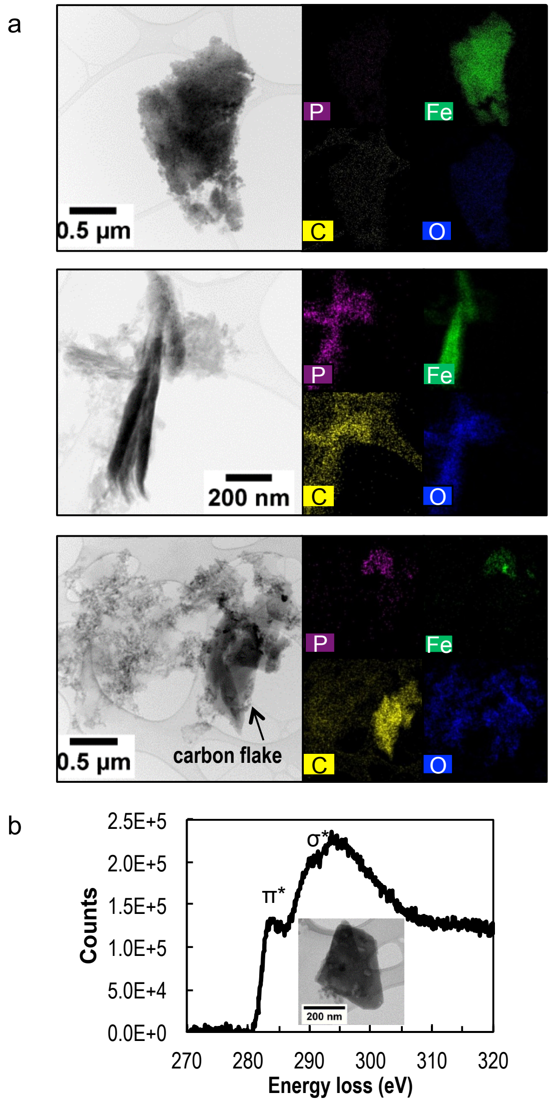

Figure S1. For a steel-DLC contact in lubrication of the base oil containing 1% [P_{66614}][DEHP], carbon debris was observed in addition to acicular debris and clusters of fine spherical nanoparticles. (a) BF-STEM images of various aggregates of wear debris particles; (b) EELS spectrum of a carbon debris. EELS spectrum indicates unfilled π* and σ* anti-bonding states for the carbon. The π* peak at 285 eV followed by the σ* peak at 291.7 eV, indicating amorphous carbon, which matches with the those reported for DLC.[1,2] This suggests the carbon debris likely scratched off from DLC surface.

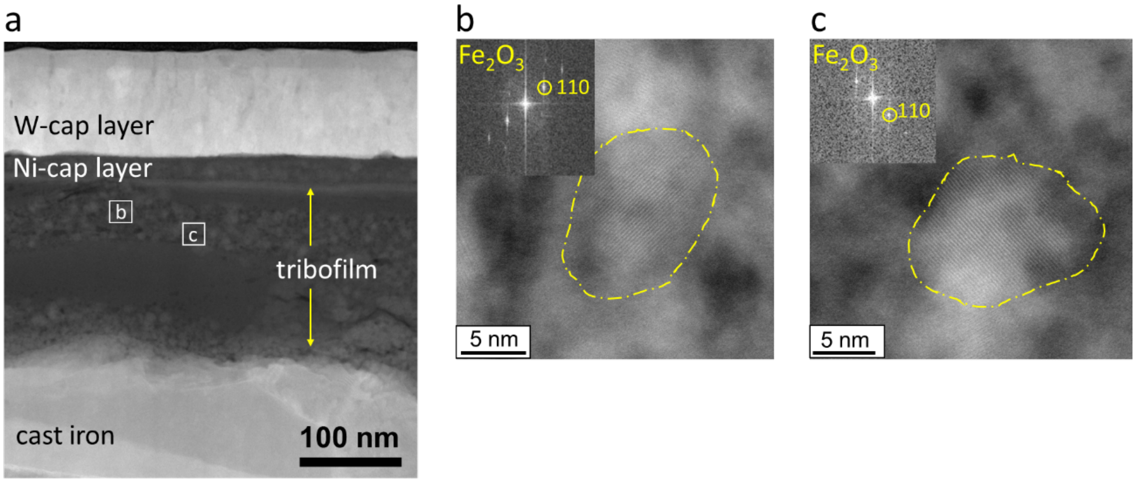

Figure S2. Wear debris nanoparticles were enclosed in a tribofilm through mechanical mixing. The tribofilm was formed on a cast iron surface by 1% [P_{8888}][DEHP] in a base oil. (a) STEM cross-sectional images of tribofilm (Note: W and Ni deposition on surface was for protection during FIB preparation); (b, c) The magnified BF images and the associated Fast Fourier Transformation (FFT) prove the existence of Fe_2O_3.

Reference

1. F. Langenhorst, V.L. Solozhenko, ATEM-EELS study of new diamond-like phases in the B-C-N system. *Phys. Chem. Chem. Phys.* 4, 5183 (2002).
2. P.K. Chu, L.H. Li, Characterization of amorphous and nanocrystalline carbon films. *Mater. Chem. Phys*. 96, 253-277 (2006).
